# The Behavior of Polyurethane Foam-Filled Glass-Fiber-Reinforced Polymer Crossarm Subjected to Lightning Transient Voltage

**DOI:** 10.3390/ma14195628

**Published:** 2021-09-28

**Authors:** Muhammad Syahmi Abd Rahman, Mohd Zainal Abidin Ab Kadir, Muhamad Safwan Abd Rahman, Miszaina Osman, Ungku Anisa Ungku Amirulddin, Shamsul Fahmi Mohd Nor, Noorlina Mohd Zainuddin

**Affiliations:** 1 Institute of Power Engineering, Universiti Tenaga Nasional, Kajang 43000, Selangor, Malaysia; asafwan@uniten.edu.my (M.S.A.R.); miszaina@uniten.edu.my (M.O.); anisa@uniten.edu.my (U.A.U.A.); shamsulfahmi.mohdnor@gmail.com (S.F.M.N.); noorlina.zainuddin@uniten.edu.my (N.M.Z.); 2 Advanced Lightning, Power and Energy Research Centre (ALPER), Universiti Putra Malaysia, Serdang 43400, Selangor, Malaysia; mzk@upm.edu.my

**Keywords:** GFRP composite crossarm, fiberglass, polyurethane foam, electric field, critical flashover voltage lightning impulse voltage, finite element method

## Abstract

The demand for composite materials in high-voltage electrical insulation is escalating over the last decades. In the power system, the composite glass-fiber-reinforced polymer has been used as an alternative to wood and steel crossarm structures due to its superior properties. As a composite, the material is susceptible to multi-aging factors, one of which is the electrical stress caused by continuous and temporary overvoltage. In order to achieve a better insulation performance and higher life expectancy, the distribution of the stresses should firstly be studied and understood. This paper focuses on the simulation work to better understand the stress distribution of the polyurethane foam-filled glass-fiber-reinforced polymer crossarm due to the lightning transient injection. A finite-element-based simulation was carried out to investigate the behavior of the electric field and voltage distribution across the sample using an Ansys Maxwell 3D. Electrical stresses at both outer and inner surfaces of the crossarm during the peak of lightning were analyzed. Analyses on the electric field and potential distribution were performed at different parts of the crossarm and correlated to the physical characteristics and common discharge location observed during the experiment. The results of the electric field on the crossarm indicate that both the outer and internal parts of the crossarm were prone to high field stress.

## 1. Introduction

**Figure 1:**
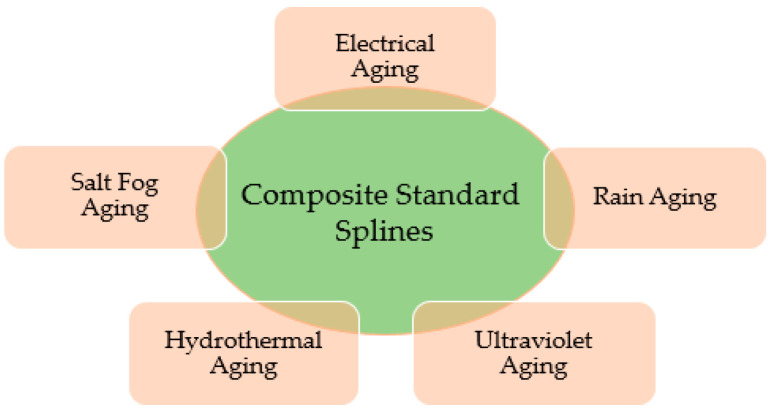
The multifactor aging of composites material [4].

Nowadays, composite materials have been used in broad applications. Glass-fiber-reinforced polymer (GFRP) composites have been used in recent decades for high-voltage (HV) applications, especially for transmission tower design to replace wooden crossarm [1]. GFRP has been recognized to have an excellent mechanical strength-to-weight ratio, corrosion resistance, longer lifespan, and cost-saving capabilities in installation and replacement, which have driven many designers to use it for electrical insulation. However, it is still vulnerable to aging, responsible for the composite’s long-term performance [2,3]. Some of the factors contributing to aging include electrical, salt fog, rain, ultraviolet (UV), and hygrothermal caused degradation as summarized in Figure 1 [4].

The extensive use of composites in HV equipment has motivated much further research on their mechanical and electrical performance as well [5,6,7,8]. The GFRP crossarm consists of a hollow tubes structure, which is a product of the pultrusion process. The process combines layers of fiberglass fabric by thermoset special polyester resin and is covered by polyurethane (PU). The hollow tube structure is typically filled with a closed-cell PU foam to eliminate the void spaces, which can cause moisture accumulation, thereby increasing the risk of electrical bridging or fires [9,10].

Previous studies evaluated the insulation performance of composite GFRP crossarm through laboratory testing to obtain AC withstand and critical flashover (CFO) voltages [11,12]. In the studies, the performance of differently aged crossarm and new crossarm were compared. At the same time, the GFRP crossarm under different conditions and polarity considering the crossarm length have been thoroughly discussed. The authors have found that the CFO per unit length of the GFRP crossarm tends to decrease as the length increased. In a separate study, the high voltage and direct current (HVDC) performance of GFRP samples were also investigated [13].

In different studies, the issue of bubble formation inside the PU foam has been discussed. The formation of the bubbles may lead to partial discharges and probably cause permanent damage to the insulation when they reach a certain threshold [14,15]. Moreover, the pore diameters of the PU foam have been inclusively studied in [14]. The authors have highlighted the challenge posed by different pore sizes that caused variation in permittivity and volume resistivity of the material, thus influencing the electrical stress distribution. The finite element method (FEM) has been widely used to evaluate the stress distribution on composite crossarms and insulators in the event of lightning [16,17,18,19]. These studies have satisfactorily predicted and located the electrical stress by its potential and E-field distributions. Simulation conducted in [20] indicated that some parts of the crossarm experienced irregular distribution of E-field along the surfaces and triple junctions. Based on the previous studies, this paper aims to understand the stress distribution and its magnitude on the external and internal parts of the GFRP crossarm, considering the lightning overvoltages.

## 2. Materials and Methods

**Figure 2:**
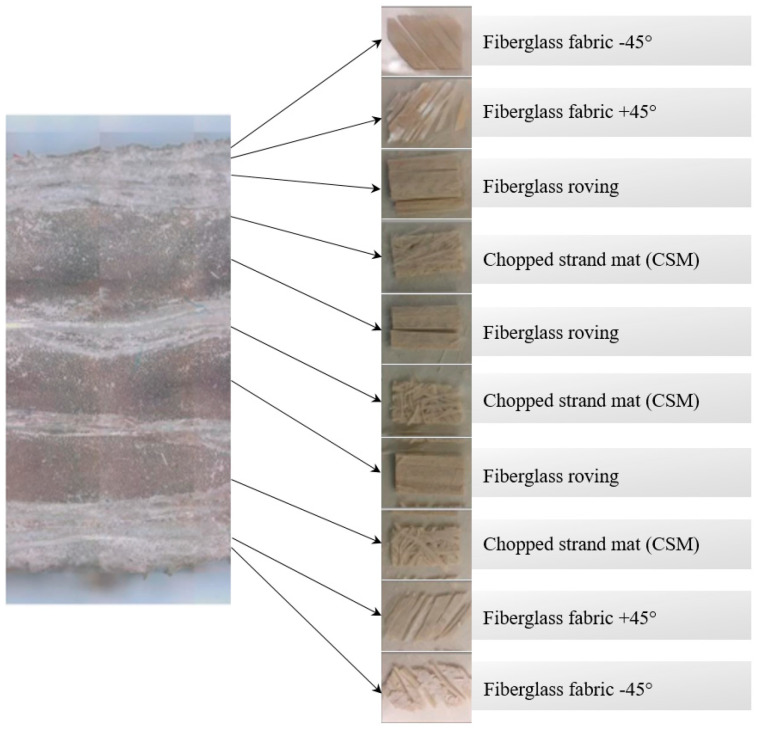
The construction layers of the GFRP crossarm sample [21].

**Figure 3:**
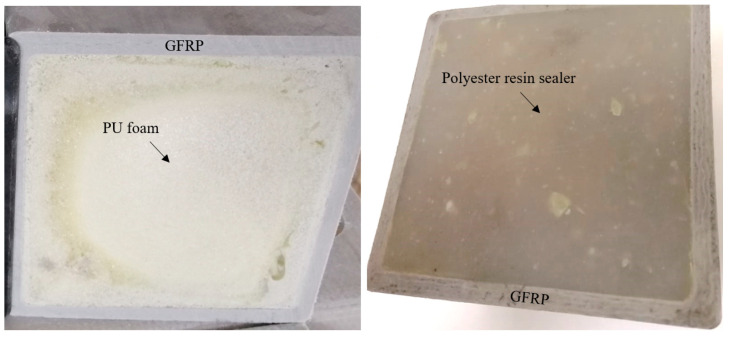
Hollow crossarm sample filled with closed-cell foam, sealed with resin sealer.

Two crossarm samples with a dimension of 100 mm × 127 mm × 127 mm were obtained from the main member of a newly pultruded 275 kV GFRP crossarm. The pultruded material comprised ten layers of fiberglass consisting of four layers of fiberglass fabric, three layers of fiberglass roving, and three layers of fiberglass chopped strand mat (CSM), with a total thickness of 7.00 mm to 7.40 mm (refer to Figure 2). The hollow samples were filled with PU foam and sealed at both ends with a special polyester resin using a cold-curing method for moisture resistance, as practiced in the field (refer to Figure 3). Later, these samples will be used as a reference for current and future studies. The methodology of the current study is divided into two sections, i.e., experimental and simulation works.

### 2.1. Experimental Work

**Figure 4:**
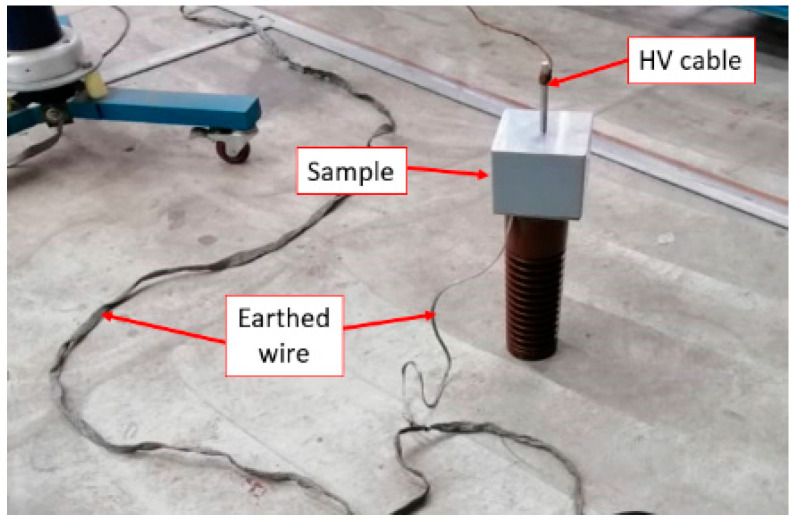
The experimental setup.

A lightning impulse test was preliminarily carried out to determine the CFO voltage of the sample by adopting the up-and-down method, as suggested in IEC 60060-1 [22]. The sample was sandwiched between two parallel plate electrodes during the test considering a uniform field, as shown in Figure 4. In total, 20 shots of standard 1.2/50 µs impulse voltage were energized at one of the electrodes, while another electrode was earthed with a 50 mΩ current shunt connected. Both positive and negative impulse polarity was considered in this study. A sample was tested three times in each polarity to investigate the loss of performance due to the repeated flashovers. The average CFO voltages obtained in the study were corrected to the standard atmospheric condition.

### 2.2. Finite Elements Simulation

**Figure 5:**
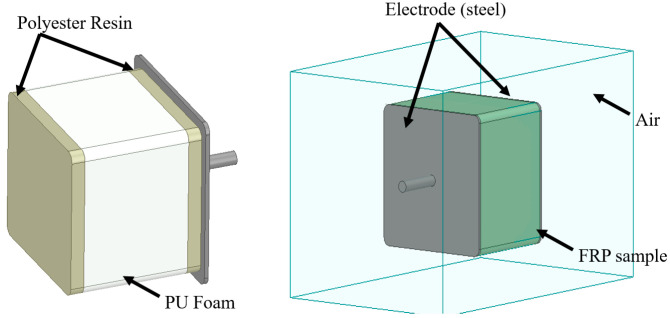
The experimental setup.

A FEM-based simulation was carried out using an Ansys Maxwell (Electronic Desktop 2020R2, Ansys Inc., Canonsburg, PA, USA) numerical package to simulate the sample’s 3-dimensional (3D) model. The 3D model, as illustrated in Figure 5, is the direct representation of the actual sample, consisting of five different materials: hollow GFRP tube, PU foam, steel electrodes, air, and polyester resin sealer. Each material was differentiated by the electrical characteristics, i.e., permittivity and bulk conductivity (S/m) as indicated in Table 1. In addition, Table 2 describes the characteristics of PU foam, which reflects the material’s permittivity and bulk conductivity.

**Figure 6:**
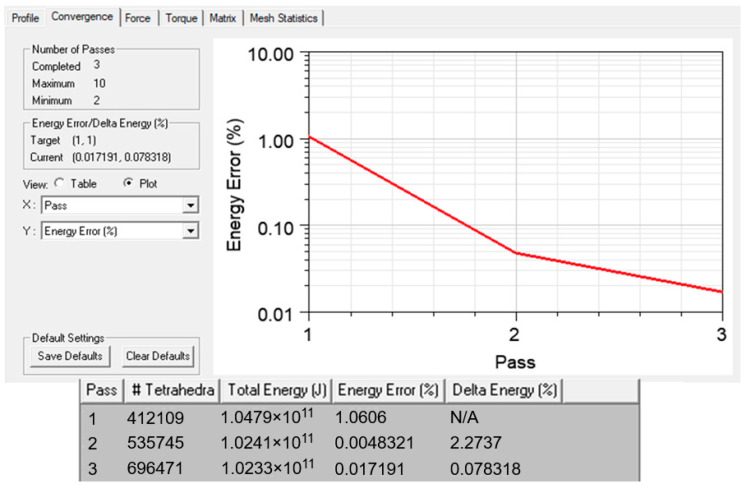
Convergence graph and iterated mesh elements.

**Figure 7:**
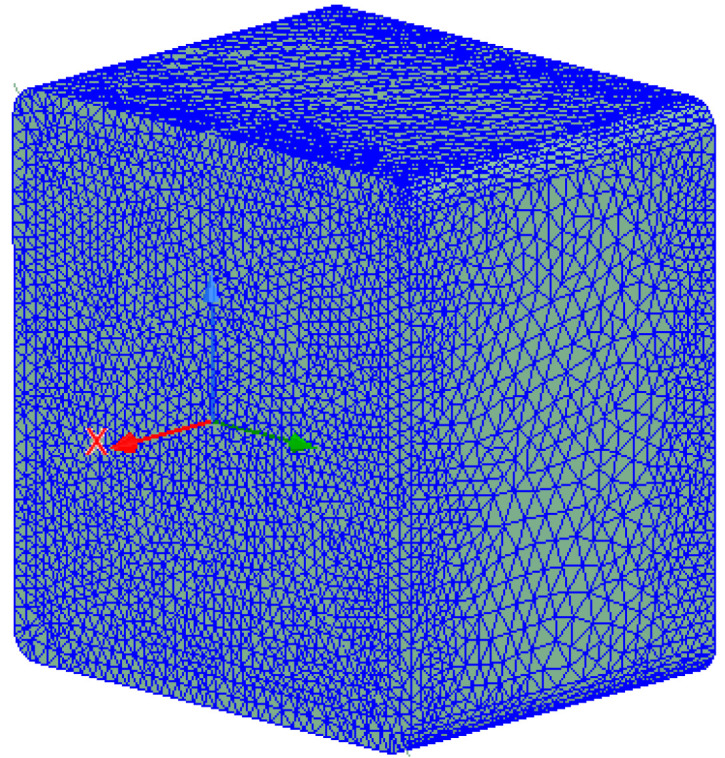
Mesh plot of the simulation model.

An optimum number of elements at 696,471 were obtained by the adaptive meshing technique, which was repeatedly refined until convergence was achieved, as shown in Figure 6. The convergence was set based on a pre-defined percentage error at 1%. Figure 7 shows the fine mesh plot of the model that was based on the finer mesh theory; a finer mesh would produce more precise and accurate results [26].

**Figure 8:**
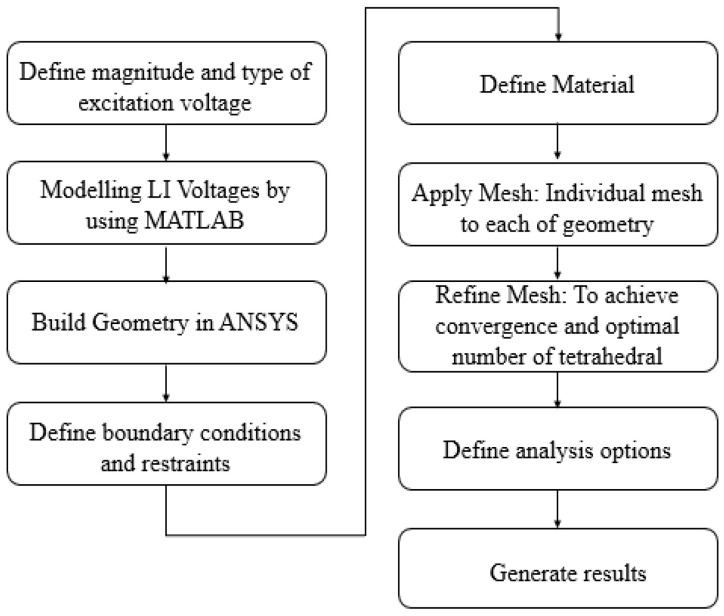
The process flow of simulation work using ANSYS.

The electric transient analysis was adopted, considering the impulse voltage in the time domain. One of the electrodes was energized with a standard lightning impulse voltage with a peak of −94.5 kV for 100 µs, which is equivalent to the CFO voltage obtained in the experiment, while another electrode was subjected to 0 kV for the same duration. The energization with CFO voltages shall reflect the maximum stress immediately before the breakdown occurs. The 1.2/50 µs lightning impulse voltage can be expressed by the double exponential function as follows [27]:

u(t) = u_o_k(℮^−αt^ − ℮^−βt^), (1)

where u_o_ is the peak value of lightning impulse voltage, and α is the attenuation coefficient of the wavefront, which is set to 1.473 × 10^4^. Meanwhile, β is the attenuation coefficient of the wave tail set to 2.08 × 10^6^, and k is the correction coefficient set to 1.043. The whole flow of the simulation study is summarized in Figure 8.

**Figure 9:**
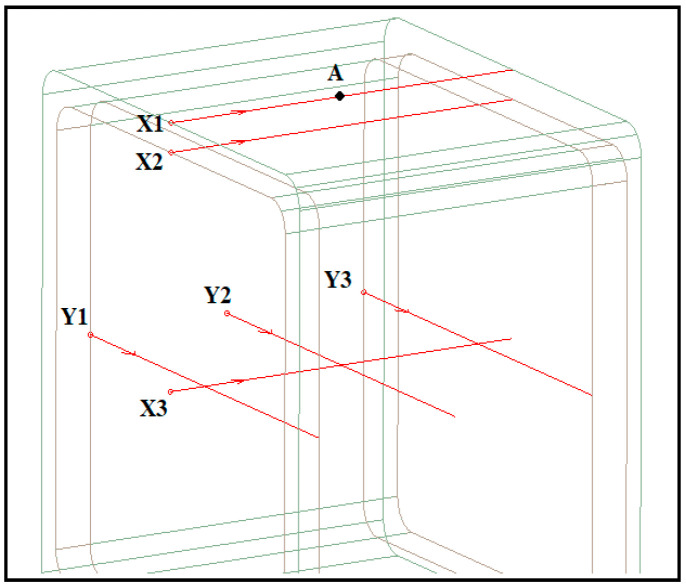
Measurement lines across the simulation model.

Several measurement lines, as illustrated in Figure 9, were introduced at different parts of the model. X1, X2, and X3 are the lines across the length (*x*-axis) laid on the outer and inner surface of GFRP and inside the foam, respectively, while Y1, Y2, and Y3 laid across the width (*y*-axis). In this study, E-field and potential distributions were generated at these lines with respect to the distance. Meanwhile, position A was introduced to investigate the variation of stress to time.

## 3. Results and Discussion

### 3.1. Critical Flashover Voltage

In the experimental works, the critical flashover voltage (CFO) of the 10 cm crossarm sample was determined at 85.2 kV and −94.5 kV for positive and negative impulse polarity, respectively. It can be found that the CFO under negative polarity was much higher than positive polarity, indicating a 10.9% difference. Investigation on the repeated test is summarized in Table 3.

**Figure 10:**
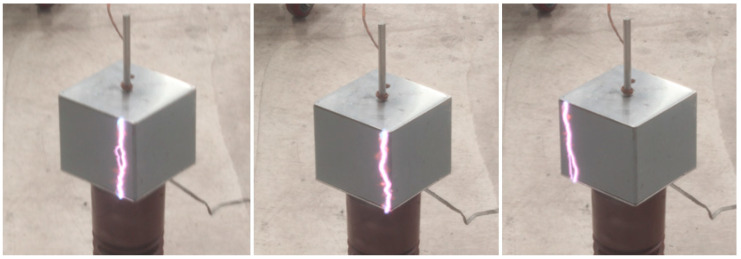
Flashover path on the surface.

It was recorded that the CFO value was slightly reduced after the subsequent test. This perhaps indicates a minor degradation of the crossarm insulation after the repeated flashovers. The observation showed that most of the flashovers occurred on the insulation surface in the air, whereas some were not visible, indicating internal flashover occurred. Most of the discharges on the surfaces happened nearer to the edges of the samples, as shown in Figure 10. For the internal flashover, it is believed that the flashover happens across the internal surface (interfaces between GFRP and foam) or within the lamination interfaces, where air voids may occasionally exist due to poor manufacturing. In this case, the non-self-healing nature of the composite is a significant concern because the post-flashover may leave conductive charred trails, yet the test conducted is not sufficient to reflect the permanent damages caused by the internal flashover.

### 3.2. FEM Analyses

**Figure 11:**
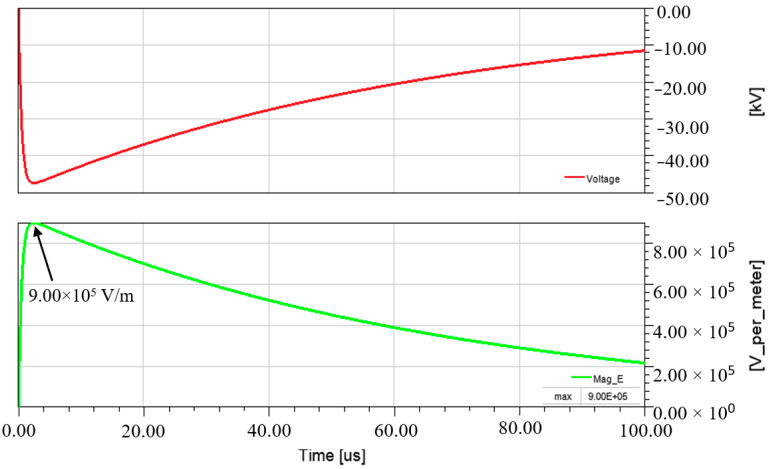
Voltage and E-field strength on GFRP surface (at position A).

Measurement of E-field strength at point A shows that the electrical stress was directly proportional to the voltage applied. The E-field profile generated at point A is illustrated in Figure 11, where the maximum E-field at 9.00 × 10^5^ V/m occurred during the peak of the lightning voltage. As the voltage reduced over time, the E-field continued to reduce relatively.

In the flashover event, the E-field on the crossarm surface was predicted to be at the maximum sufficient to sustain an arc across the insulation surface. Based on the theory of the uniform field, flashover could occur in the air at 30 kV/cm, which the threshold might vary according to the surrounding conditions [28]. Moreover, the E-fields distribution within the material is similarly important. Flashover might occur internally when the E-field exceeds the breakdown strength of the material.

**Figure 12:**
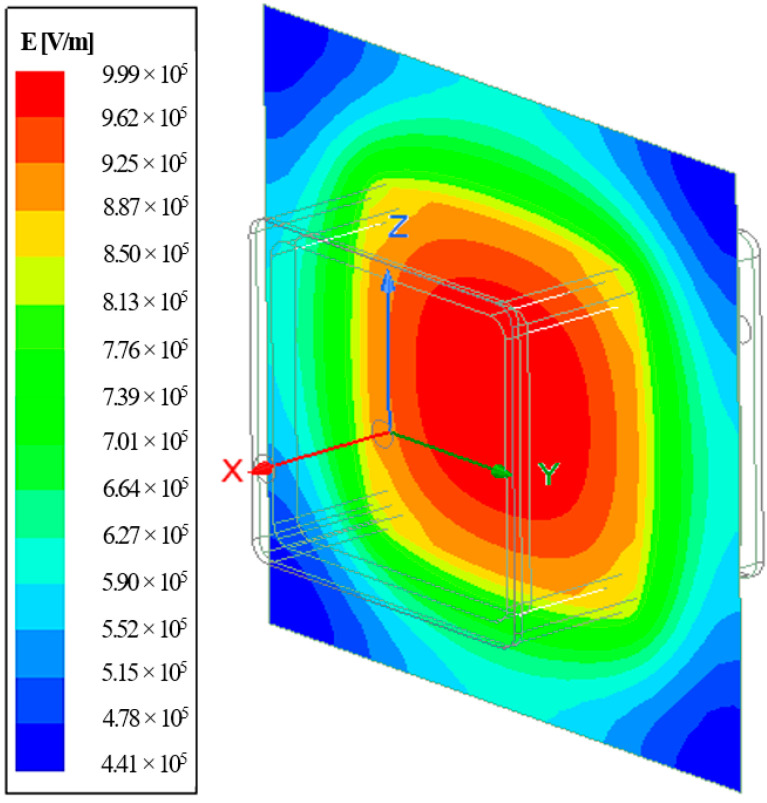
E-field distribution at the cross section of the sample.

In the current study, the conducted simulation had satisfactorily predicted E-field at the cross section of the crossarm, as illustrated in Figure 12. It showed that the field stress was abnormally distributed around the structure, which was anticipated to be evenly distributed based on the theory of parallel plate distribution. The highest E-field was primarily concentrated inside the foam up to 9.99 × 10^5^ V/m and gradually decreased away from the center.

**Figure 13:**
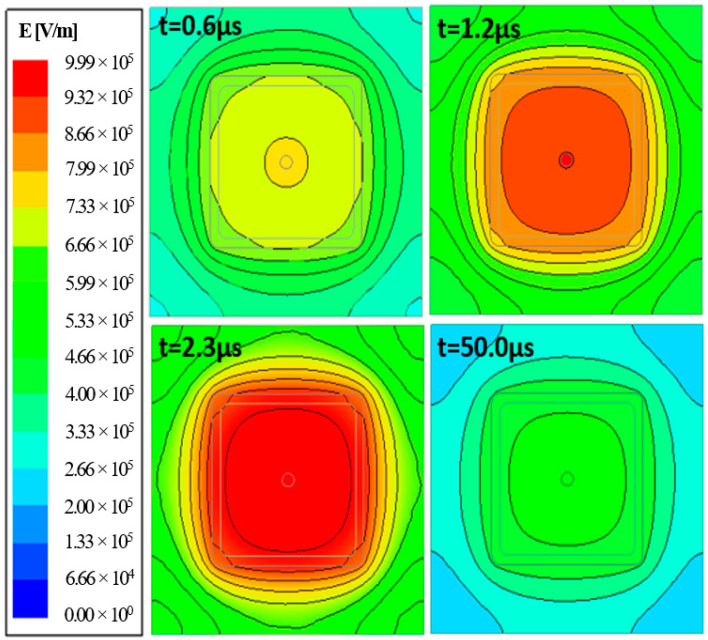
E-field at different time moments.

Investigation of the E-field distribution at different time moments of the lightning transient revealed that the E-fields on the crossarm developed from the center of the foam outwards. As illustrated in Figure 13, the maximum E-field could be observed as early as 1.2 µs (front time) and continued to expand to the entire foam region until time equal to 2.3 µs.

**Figure 14:**
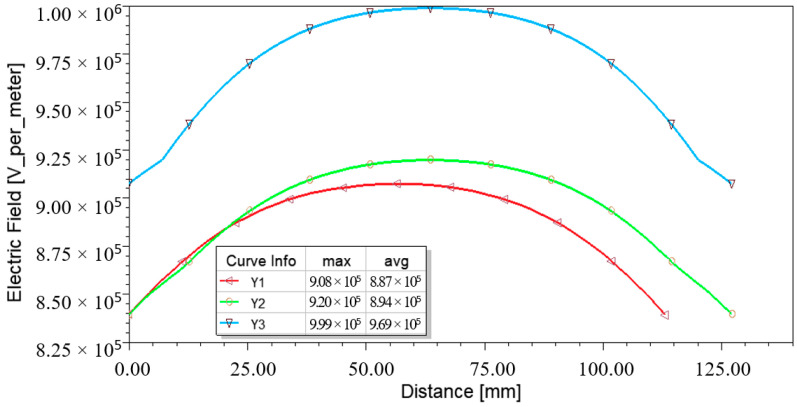
E-field profiles across Y1, Y2, and Y3 during the peak of impulse voltage.

Translated into curves, the sample’s E-field measurements across the *y*-axis (or *y* cross section) were labeled Y1, Y2, and Y3 and represented by a “dome shape” (refer to Figure 14). The profiles showed that the maximum E-field on the outer surface of GFRP was slightly lower than the internal surface, marking a 1.32% difference. In contrast, 10.02% of the difference was measured between the outer surface and foam. In general, it could be observed that the magnitude of the E-field presented in Figure 14 exceeded the typical streamer threshold that is usually initiated at a magnitude of 0.50 MV/m to 0.60 MV/m [29,30,31].

**Figure 15:**
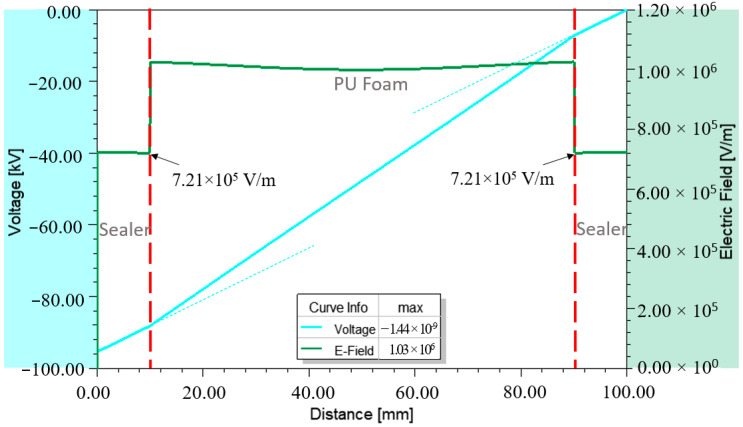
Voltage distribution versus E-field distribution across X3.

In general, the E-field intensity is closely associated with the voltage gradient across the insulating material. For example, the voltage distribution across X3 is presented in Figure 15. The voltage was non-linearly distributed across the distance where the gradient changes could be seen at 10 mm and 90 mm at which the interface of sealer and foam material existed.

**Figure 16:**
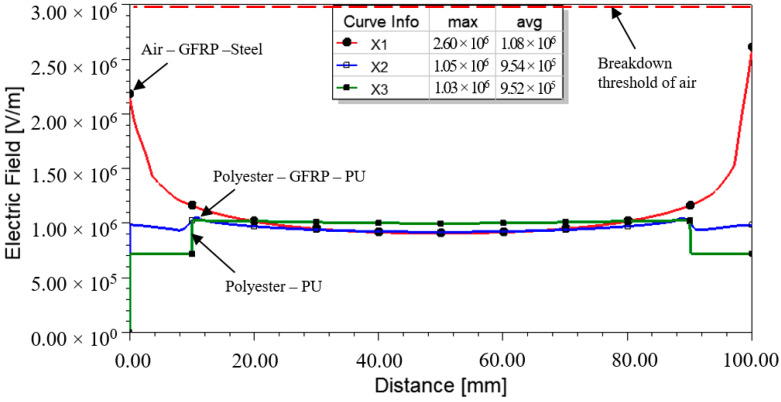
E-field profiles across X1, X2, and X3 during the peak of voltage.

Based on Figure 16, three distinct E-field profiles could be found across X1, X2, and X3. It showed that the non-homogenously distributed fields caused higher electrical stress on both ends of the samples. A maximum field up to 2.60 × 10^6^ V/m was recorded on the surface (X1) at which a triple junction between air, GFRP, and steel electrodes existed. It should be noted that this value was proximate to the breakdown threshold of air. In addition, the triple junction between polyester sealer, GFRP, and PU foam showed a minor impact on the field distribution at X2.

A maximum field up to 1.03 × 10^6^ V/m was projected across the PU foam (X3). As a comparison, this value was one-sixth of the average lightning breakdown strength of a typical closed-cell foam, which occurs typically at 6.09 × 10^6^ V/m [14]. It is unlikely for a flashover to happen across the foam material at this level. However, it should not be disregarded as the indicated value might promote partial discharges that lead to the progressive aging of the material, as the value surpassed the threshold of streamer development.

**Figure 17:**
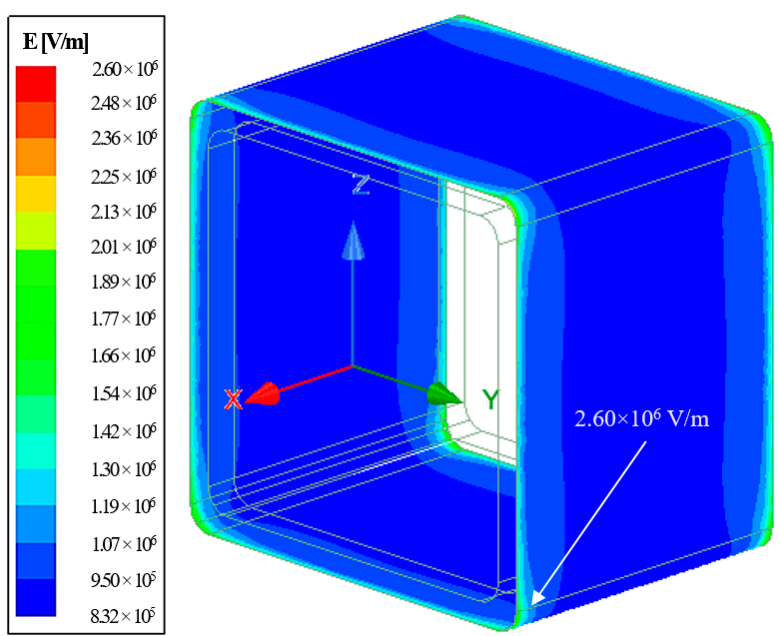
E-field stress on the external surfaces of GFRP crossarm sample.

Field plot on the outer surface of the crossarm sample revealed that the higher E-field stress was confined at every corner, as shown in Figure 17, whereby the highest E-field on the surface was 2.60 × 10^6^ V/m. The field distribution explained why most of the flashover occurred at the corners, as revealed in Figure 10.

**Figure 18:**
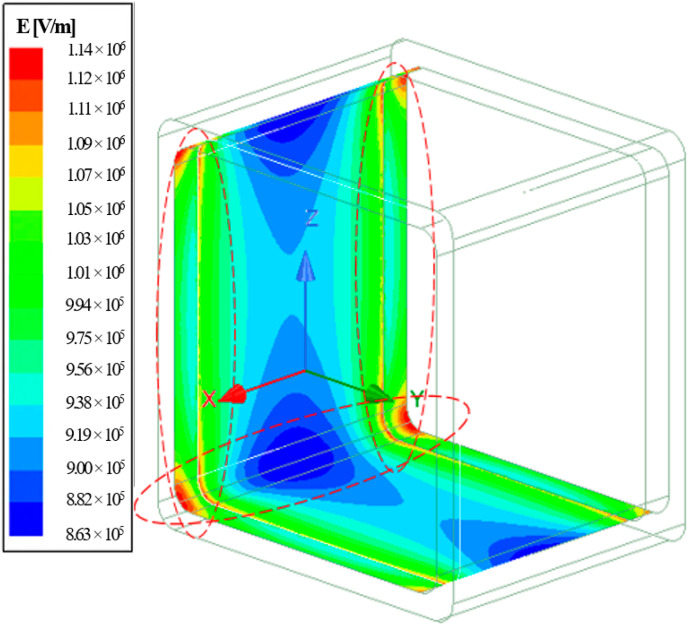
E-field stress on the internal surfaces of GFRP crossarm sample.

Similarly, the internal GFRP surface indicated the exact behavior, whereby localized E-field was more significant at all corners, having a maximum E-field up to 1.14 × 10^6^ V/m (see Figure 18). Circled in red is the material interfaces with localized E-field.

## 4. Conclusions

The experimental works successfully determined the CFO voltage of the 10 cm crossarm samples at 85.2 kV and −94.5 kV, where the polarity effect caused 10.9% of the difference. In addition, a reduction trend of CFO voltages was observed when the crossarm was subjected to a repeated lightning impulse test. It is believed that minor degradation occurred due to the electrical arc.

Analysis of the E-field distribution was carried out at different parts of the sample and correlated to the physical characteristic, and common discharge location was observed during the experiment. The FEM analysis revealed that the external GFRP surface suffered maximum electrical stress at both ends, which was consistent with the location of triple junctions. However, it significantly reduced toward the center of the sample, forming a U-shape profile distribution. Internally, the stress was found slightly lower at both sample ends due to the presence of sealer.

A dome-shape distribution could be observed across the width (*y*-axis) of the sample, where the maximum field could be found at the center. Therefore, it revealed that the middle span of the sample suffered the highest field in the PU foam, followed by the internal and external surface of GFRP. Meanwhile, the overall analysis of the sample showed that the edges suffered the highest E-field, which is understood to be caused by the pointed shape and the triple junctions, correlated to the flashover arc’s location.

In the current study, the way in which the composite material reacts to the E-field was therefore considered to understand their long-term performance. It was also suggested that fair attention should be given to the insulation properties of the foam during designing since the E-field that developed during the lightning transient exceeded the typical threshold of streamers. Even though the localized E-field is not responsible for an instant loss of insulation, it may lead to partial discharges that might cause a preliminary fault in the crossarm and degradation over some time. By knowing the magnitude of stresses on the crossarm, an appropriate selection of insulation material, properties, shapes, etc. can be planned to keep stress at a minimum level.

## Figures and Tables

**Table 1:** Material-based parameters.

Parts	Material	Relative Permittivity (ɛ_r_)	Volume Conductivity (σ) S/m	Ref.
Air	Air	1.0006	0	Ansys Lib.
Electrodes	Steel	1.0	2.00 × 10^6^	Ansys Lib.
Crossarm	GFRP	4.6	1.11 × 10^−14^	[23,24]
Foam	Polyurethane	1.8	5.56 × 10^−15^	[25]
Sealer	Polyester resin	3.2	0	Ansys Lib.

**Table 2:** Characteristics of PU foam used in the simulation [25].

Characteristics	Description
Pore Geometry	Spherical
Density, g/cm^3^	0.2510
Average Diameter, µm	200
Pore Porosity, %	73

**Table 3:** CFO voltages of crossarm sample.

Polarity	Sample	CFO Voltage, U_50_ (kV)	Standard Deviation (± %)	No. of Withstand	No. of Flashover
Positive	Sample 1	85.2	3.43	10	10
		84.6	2.43	9	11
		83.9	2.84	8	12
Negative	Sample 2	−94.5	1.47	10	10
		−93.7	1.81	10	10
		−92.6	1.59	9	10

## Data Availability

Not applicable.

## References

[1] Rawi I.M., Abd Rahman M.S., Ab Kadir M.Z.A., Izadi M. Wood and fiberglass crossarm performance against lightning strikes on transmission towers. Proceedings of the International Conference on Power System.

[2] Yang B., Zhang J., Zhou L., Lu M., Liang W., Wang Z. (2015). Effect of fiber surface modification on water absorption and hydrothermal aging behaviors of GF/pCBT composites. Compos. Part B.

[3] Mlyniec A., Korta J., Kudelski R., Uhl T. (2014). The influence of the laminate thickness, stacking sequence and thermal aging on the static and dynamic behavior of carbon/epoxy composites. Compo. Struct..

[4] Shao J., Wang J., Long M., Li J., Ma Y. (2017). 5000 h multi-factor accelerated aging test of frp made transmission tower: Characterization, thermal decomposition and reaction kinetics study. Polymers.

[5] Asyraf M.R.M., Ishak M.R., Sapuan S.M., Yidris N. (2021). Utilization of Bracing Arms as Additional Reinforcement in Pultruded Glass Fiber-Reinforced Polymer Composite Cross-Arms: Creep Experimental and Numerical Analyses. Polymers.

[6] Mohamad D., Syamsir A., Beddu S., Abas A., Ng F., Razali M., Seman S.A.H.A. (2019). Numerical study of composite fiberglass cross arms under statics loading and improvement with sleeve installation. IOP Conf. Ser. Mater. Sci. Eng..

[7] Sá M.F., Gomes A.M., Correia J.R., Silvestre N. (2011). Creep behavior of pultruded GFRP elements–Part 1: Literature review and experimental study. Compos. Struct..

[8] Xin H., Liu Y., Mosallam A.S., He J., Du A. (2017). Evaluation on material behaviors of pultruded glass fiber reinforced polymer (GFRP) laminates. Compos. Struct..

[9] Britt W.F. (2017). Composite Structural Support Arm. U.S. Patent.

[10] Pieper R.J. (2016). The use of mechanical testing, photomicrography an electron microscopy to characterize an insulating fiberglass composite post-electrical arc failure. Microsc. Microanal..

[11] Grzybowski S. Electrical performance of 115 kV transmission lines fiberglass crossarms aged in field. Proceedings of the 5th International Conference on Properties and Applications of Dielectric Materials.

[12] Grzybowski S., Disyadej T. Electrical performance of fiberglass crossarm in distribution and transmission lines. Proceedings of the 2008 IEEE/PES Transmission and Distribution Conference and Exposition.

[13] Laninga J., Amer M., Swatek D., McDermid W., Kordi B. HVDC flashover performance of fibreglass reinforced (FRP) hot sticks considering space charges. Proceedings of the 2017 IEEE Conference on Electrical Insulation and Dielectric Phenomenon (CEIDP).

[14] Karady G.G., Argin M., Shi B., Rahmatian F., Rose A.H. Electrical properties of rigid pour polyurethane foam applied for high voltage insulation. Proceedings of the 2003 IEEE PES Transmission and Distribution Conference and Exposition (IEEE Cat. No. 03CH37495).

[15] Strauchs A., Mashkin A., Schnettler A., Podlazly J., Freiheit-Jensen B. Investigations on the partial discharge behavior of syntactic foam under uniform field stress. Proceedings of the IEEE International Symposium on Electrical Insulation.

[16] Abd Rahman M.S., Ab Kadir M.Z.A., Ab-Rahman M., Osman M., Nor S.F.M. Lightning impulse strength of 275 kV and 132 kV Tower with composite crossarm. Proceedings of the 11th Asia-Pacific International Conference on Lightning (APL).

[17] Abd Rahman M.S., Ab Kadir M.Z.A., Ab-Rahman M., Osman M., Nor S.F.M., Mohd Zainuddin N. (2020). Effects of a Crossarm Brace Application on a 275 kV Fiberglass-Reinforced Polymer Crossarm Subjected to a Lightning Impulse. Energies.

[18] Jahangiri T., Wang Q., Bak C.L., da Silva F.F., Skouboe H. (2017). Electric stress computations for designing a novel unibody composite cross-arm using finite element method. IEEE Trans. Dielectr. Electr. Insul..

[19] Shanmugam G., Karakkad S. (2018). Influence of the insulator geometry on the streamer propagation characteristics in polymeric insulators under positive polarity lightning impulse voltages. IET Sci. Meas. Technol..

[20] Zachariades C., Rowland S.M., Cotton I., Peesapati V., Chambers D. (2015). Development of electric-field stress control devices for a 132 kV insulating cross-arm using finite-element analysis. IEEE Trans. Power Deliv..

[21] Rosli A.N. (2020). Numerical Modelling of Glass Fiber Reinforced Polymer (GFRP) Crossarm in Transmission Tower Subjected to Static Loading. Master’s Dissertation.

[22] (2010). IEC60060-1: High Voltage Test Techniques—Part 1: General Definition and Test Requirements.

[23] Tuncer E., Sauers I., James D.R., Ellis A.R. (2009). Electrical insulation characteristics of glass fiber reinforced resins. IEEE Trans. Appl. Supercond..

[24] Yamano Y., Takahashi M., Kobayashi S., Hanada M., Ikeda Y. (2008). Surface discharge related properties of fiberglass reinforced plastic insulator for use in neutral beam injector of JT-60U. Rev. Sci. Instrum..

[25] Liang X., Shen Y., Liu Y., Wang J., Gao Y., Li S., Wang M., Gao S. Investigations on the basic electrical properties of Polyurethane foam material. Proceedings of the 2015 IEEE 11th International Conference on the Properties and Applications of Dielectric Materials (ICPADM).

[26] Huang D., Ruan J., Chen Y., Huo F., Yu S., Liu S. Calculation and measurement of potential and electric field distribution along 1000 kV AC transmission line composite insulator. Proceedings of the ICEMS 2008: International Conference on Electrical Machines and Systems.

[27] Yang X., Wang Q., Wang H., Zhang S., Peng Z. (2016). Transient electric field computation for composite cross-arm in 750 kV AC transmission line under lightning impulse voltage. IEEE Trans. Dielectr. Electr. Insul..

[28] Ryan H.M. (2013). Fundamental aspects of air breakdown. High Voltage Engineering and Testing.

[29] Shanmugam G., Samajdar G., Karakkad S. Surface Charging and its Influence on Lightning Impulse Flashover Characteristics of Polymeric Insulator. Proceedings of the 2019 IEEE International Conference on Electrical, Computer and Communication Technologies (ICECCT).

[30] Thione L., Pigini A., Allen N., Aro M., Baker A. (1992). Guidelines for the Evaluation of the Dielectric Strength of External Insulation.

[31] Liu L., Becerra M. (2017). An efficient model to simulate stable glow corona discharges and their transition into streamers. J. Phys. D Appl. Phys..

